# Assessing effectiveness of ABCDE Framework for teaching condylar fracture reduction in dental education: a mixed methods study

**DOI:** 10.1186/s12909-025-07705-7

**Published:** 2025-07-31

**Authors:** Jun Pang, Zhigan Lv, Haifeng Zhang, Siyao Yang, Yuanyuan Wang, Yang Wu, Xiaoxing Hao, Lin Cheng, Pengfei Xin

**Affiliations:** 1 Department of Anesthesiology, Shanxi Bethune Hospital, Shanxi Academy of Medical Sciences, Tongji Shanxi Hospital, Third Hospital of Shanxi Medical University, Taiyuan, 030032 China; 2 Health Examination Department, Shanxi Bethune Hospital, Shanxi Academy of Medical Sciences, Tongji Shanxi Hospital, Third Hospital of Shanxi Medical University, Taiyuan, 030032 China; 3 Department of Stomatology, Shanxi Bethune Hospital, Shanxi Academy of Medical Sciences, Tongji Shanxi Hospital, Third Hospital of Shanxi Medical University, Taiyuan, 030032 China

**Keywords:** ABCDE framework, Condylar fracture, Dental education, Reduction and fixation, Standardized residency training, Technology Acceptance Model

## Abstract

**Background:**

Managing displaced proximal segments in mandibular condylar fractures remains challenging due to complex anatomy and limited surgical visibility. Conventional teaching methods in oral and maxillofacial surgery (OMFS) often inadequately address these challenges. This study evaluates the effectiveness of a novel ABCDE educational framework, based on the Technology Acceptance Model (TAM), in enhancing surgical training by improving procedural understanding, technical skill acquisition, and trainee confidence in managing these fractures.

**Methods:**

The ABCDE framework includes Assessment, Briefing, Collaborative learning, Demonstration, and Evaluation phases. Participants were 39 dental residents (Grades 1–3) enrolled in a 4-month OMFS rotation as part of their clinical residency program. Due to staggered rotation schedules, not all participants had participated in surgical rotations for condylar fracture reduction by the time of the study. All participants completed a two-day training program combining lectures and hands-on sessions. A convergent parallel mixed-methods design was used: quantitative assessments via pre- and post-intervention tests; qualitative analysis of open-ended questionnaires via thematic coding. Statistical analyses were conducted in RStudio using R software.

**Results:**

Thirty-six participants completed pre- and post-intervention tests and questionnaires, while three did not participate in assessments. Quantitative assessments were conducted using pre- and post-intervention tests, while qualitative analysis was performed on open-ended questionnaires using thematic coding techniques. Following the intervention, students’ test scores demonstrated a significant improvement (median 25.00 [95% confidence interval (CI): 23.75, 30.00] to median 30.00 [95% CI: 25.00, 35.00], p < 0.01). Over 70% of residents reported positive satisfaction and learning gains, with correspondence analysis confirming strong associations between these outcomes. The Net Promoter Score (NPS) for the ABCDE framework was 44.44, indicating moderate-to-high advocacy. Qualitative insights emphasized its practicability, ease of use, and ability to boost engagement and skill acquisition.

**Conclusions:**

The ABCDE framework bridges theoretical knowledge and hands-on practice, addressing anatomical and technical challenges in teaching mandibular condylar fracture management. Its TAM-driven modular design prioritizes perceived usefulness and perceived ease of use, addressing conventional teaching limitations. Resident feedback emphasized increased satisfaction and advocacy, underscoring the value of interactive, teamwork-focused strategies in medical training.

**Supplementary Information:**

The online version contains supplementary material available at 10.1186/s12909-025-07705-7.

## Background

The surgical management of medially displaced proximal segments in mandibular condylar fractures is highly challenging due to spatial constraints imposed by the lateral pterygoid muscle, facial nerve, branches of the external carotid artery, and adjacent temporomandibular joint (TMJ) bony structures [1, 2]. These anatomical limitations significantly restrict visibility and surgical maneuverability during reduction, making effective reduction and stabilization prior to fixation critical for successful outcomes [1]. To address these challenges, various techniques have been proposed, including bone-holding forceps, curved artery forceps, Moule pins [3], screws [4, 5], and titanium plates [6]. Additionally, downward traction of the distal segment (ramus) is essential for repositioning the proximal segment into the glenoid fossa.

In clinical practice, several methods are employed to achieve downward ramus traction for condylar fracture reduction [2, 6–17]. Given the complexity and diversity of these techniques, a summary table (Table 1) is provided to clarify their indications, operational key points, tools/materials and characteristics. While the “hole and wire” method is no longer listed on the AO website, it remains referenced in historical literature [8, 9], and may be applicable in specific low-resource or complex cases where alternative techniques are unavailable.

**Table 1:** Summary of techniques for downward ramus traction in condylar fracture reduction

No	Technique	Operation key points	Tools/Materials	Characteristics	References
1	Classic manual traction	Thumbs wrapped in gauze apply downward pressure on molars/retromolar area to reposition condyle	None (manual force)	Aligns with traditional Hippocratic approach; no additional tools required but relatively labor-intensive	[7]
2	Twisted steel wire traction	1. Wire looped around screws in lateral mandibular ramus 2. Wire inserted through drilled holes at inferior mandibular angle	Steel wire, drill, screws	Provides stable bony anchorage; minimizes soft tissue trauma; suitable for significantly displaced fractures	1. [2, 6] 2. [8, 9]
3	Forceps traction	Forceps applied to drilled holes in masseteric tuberosity or directly clamped onto the mandible for percutaneous traction	Drape forceps, Eckelt/Rasse fracture forceps	Requires careful handling to avoid soft tissue laceration from instrument slippage; Utilizing the pull-and-release maneuvers with forceps	[10, 11]
4	Hook traction	Gillis or Hugonnier hook placed at mandibular notch to pull ramus downward	Gillis/Hugonnier hook	Provides reliable mechanical leverage for high or medially displaced condylar fractures; Requires caution to avoid injury to neurovascular structures medial to the mandibular ramus; Contraindicated in cases with associated mandibular notch fractures	[10]
5	Retractor devices	Specialized retractors (e.g., custom mandibular joint retractor) inserted to maintain reduction	Custom retractor with wrench system	Sustained traction for stable visualization during reduction and fixation	[12]
6	Heister mouth gag	Gag arms rest on zygomatic arch and distal fragment to distract ramus	Heister mouth gag	Contraindicated in cases with zygomatic arch fractures	[13]
7	Occlusal splint	Custom occlusal splint to maintain occlusion and traction	Occlusal splint, elastic bands	Suitable for intraoral approaches; preserves occlusal relationship during reduction	[14]
8	Transoral endoscopic-assisted approaches	1. Sterile silastic wedge between posterior teeth with intermaxillary fixation 2. An auto reposition and fixation osteosynthesis plate 3. Ulm protocol: template-guided patient-specific osteosynthesis implant	Silastic block or individualized implant design	Endoscopic equipment required, no facial skin incision, and almost no risk of facial nerve injury	1. [15] 2. [16] 3. [17]

Conventional instructor-led lectures remain valuable for maintaining student satisfaction and foundational theoretical instruction but demonstrate limitations in fostering active learning, skill application, and efficient management of complex cases [18–20]. In our experience with the oral and maxillofacial surgery (OMFS) curriculum, teaching of condylar fracture reduction similarly encounters these limitations. Students often struggle to integrate anatomical knowledge with the technical demands of surgery, particularly when real-time adjustments are required during procedures. Even for relatively straightforward cases such as subcondylar fractures, variations in clinical experience among practitioners—including those already in practice—can lead to hesitation or uncertainty in decision-making and technique [21]. Furthermore, the current education system faces the persistent challenge of the theory–practice gap, underscoring the need to implement strategies such as developing “clinically culture-based content” and a “clinically based curriculum” to bridge this divide [22]. Additionally, student engagement is often an elusive goal, and it is important for instructors to combine collaboration, open-ended exploration, and problem-based learning in real-life scenarios into a single project that works flexibly across disciplines and institutions [23]. While the current implementation of virtual reality (VR) in dental education shows promise, particularly in enhancing students’ engagement, more comprehensive and robust research is needed to validate its effectiveness fully, and trainers did not fully support replacing conventional pre-clinical training methods [24].

To address the limitations of traditional instruction in OMFS, innovative strategies such as flipped operating room techniques, case-based learning (CBL), and hands-on training have emerged as promising solutions [25–27]. These approaches aim to enhance learner engagement and contextual understanding while complementing, rather than replacing, established teaching methods. Building on these strategies, this study proposes a structured educational framework (the ABCDE framework) designed to improve comprehension of condylar fracture reduction without requiring advanced tools such as VR equipment. The framework integrates step-by-step procedural simulations and team-based case scenarios to directly address key shortcomings of conventional instruction.

Grounded in the Technology Acceptance Model (TAM) [28], the framework emphasizes *perceived usefulness* and *ease of use* to foster learner engagement. TAM has been widely used in educational research to explain how users accept and interact with new learning technologies [29]. Additionally, the Net Promoter Score (NPS), a validated metric increasingly adopted in continuing medical education and higher education [30, 31], to assess learner satisfaction and loyalty. By categorizing learners into promoters, passives, and detractors, NPS offers actionable feedback on the impact of educational interventions on skill development and learner confidence. NPS is used in this study to gauge the *perceived usefulness* and *perceived ease of use* of the framework, thereby providing insights into its effectiveness in fostering learner engagement and skill acquisition. Accordingly, the following hypothesis was formulated:

Hypothesis 1: Perceived usefulness of the ABCDE framework positively predicts NPS satisfaction (i.e., promoters), aligning with TAM’s core tenets.

## Methods

### Study design and participants

This study employed a convergent parallel mixed-methods research design [32], integrating both quantitative and qualitative data to comprehensively evaluate the effectiveness of an educational intervention aimed at improving dental residents’ knowledge and skills in managing condylar fractures. Specifically, this design allowed simultaneous collection and analysis of pre- and post-intervention assessments (quantitative) alongside participant feedback surveys (qualitative), enabling triangulation of findings to enrich interpretation. The study involved all 39 standardized dental residents at Shanxi Bethune Hospital’s Department of Stomatology. These residents are part of the comprehensive oral medicine training program (oral general practitioners) in China, undergoing structured residency training rather than being employed as hospital staff. All 39 residents were enrolled in the 4-month OMFS training program as per the standardized curriculum. However, the timing of their rotations varied, with some Grade 1 and Grade 2 residents not yet having commenced their OMFS rotations at the time of this study, while Grade 3 residents had already completed their rotations. Surgical exposure to condylar fractures was subgroup-dependent (e.g., tumor or trauma rotations), and pre-intervention surgical experience was unknown a priori. All participants underwent identical training in small group settings. For anonymity, a streamlined identification system using *DeepSeek* generated 39 familiar four-letter words (e.g., ‘book’, ‘tree’, ‘flow’), allowing students to select unique codes from this verified word bank, ensuring privacy while matching pre-and post-survey responses.

### Educational framework

**Fig. 1:**
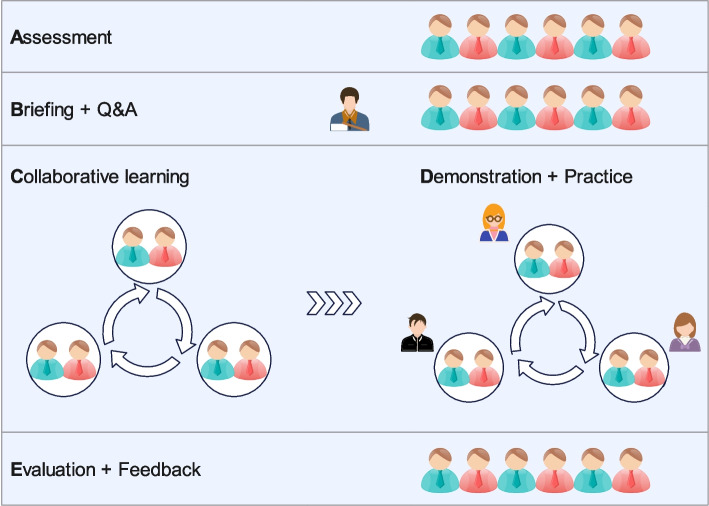
Schematic representation of the ABCDE educational framework

The intervention was structured around the ABCDE framework: Assessment, Briefing, Collaborative learning, Demonstration, and Evaluation (Fig. 1). This learner-centered approach was designed to scaffold knowledge acquisition and skill development through iterative engagement with theoretical content and hands-on practice.

- Assessment: Baseline knowledge on condylar fracture reduction was evaluated using a validated questionnaire to identify knowledge gaps and inform instructional design.
- Briefing: An experienced instructor delivered foundational lectures supplemented with schematic diagrams, focusing on mandibular anatomy and various reduction techniques.
- Collaborative learning: Students were divided into small groups to research and discuss specific reduction categories, fostering peer-to-peer learning and critical thinking.
- Demonstration: Faculty demonstrated each reduction technique using anatomical models and simulation tools. Students practiced these procedures under supervision, emphasizing key steps and techniques.
- Evaluation: Formative assessments included peer evaluations during group discussions where representatives demonstrated surgical skills, receiving feedback from peers and instructors. Summative assessments evaluated overall learning outcomes at the end of the intervention.

### Reduction techniques

Reduction techniques were categorized into three groups:

- Category I (Proximal segment reduction): Involves placing titanium screws, plates, or Moule pins in the proximal segment for stable fixation.
- Category II (Distal segment traction with wires): Uses twisted wires either looped around screws implanted laterally on the ramus or inserted into drilled holes at the inferior border of the mandibular angle.
- Category III (Alternative methods): Includes classic manual traction, hook use, retractor application, and interdental blocks, focusing on alternative tools for aiding condylar segment reduction.

### Educational materials

**Fig. 2:**
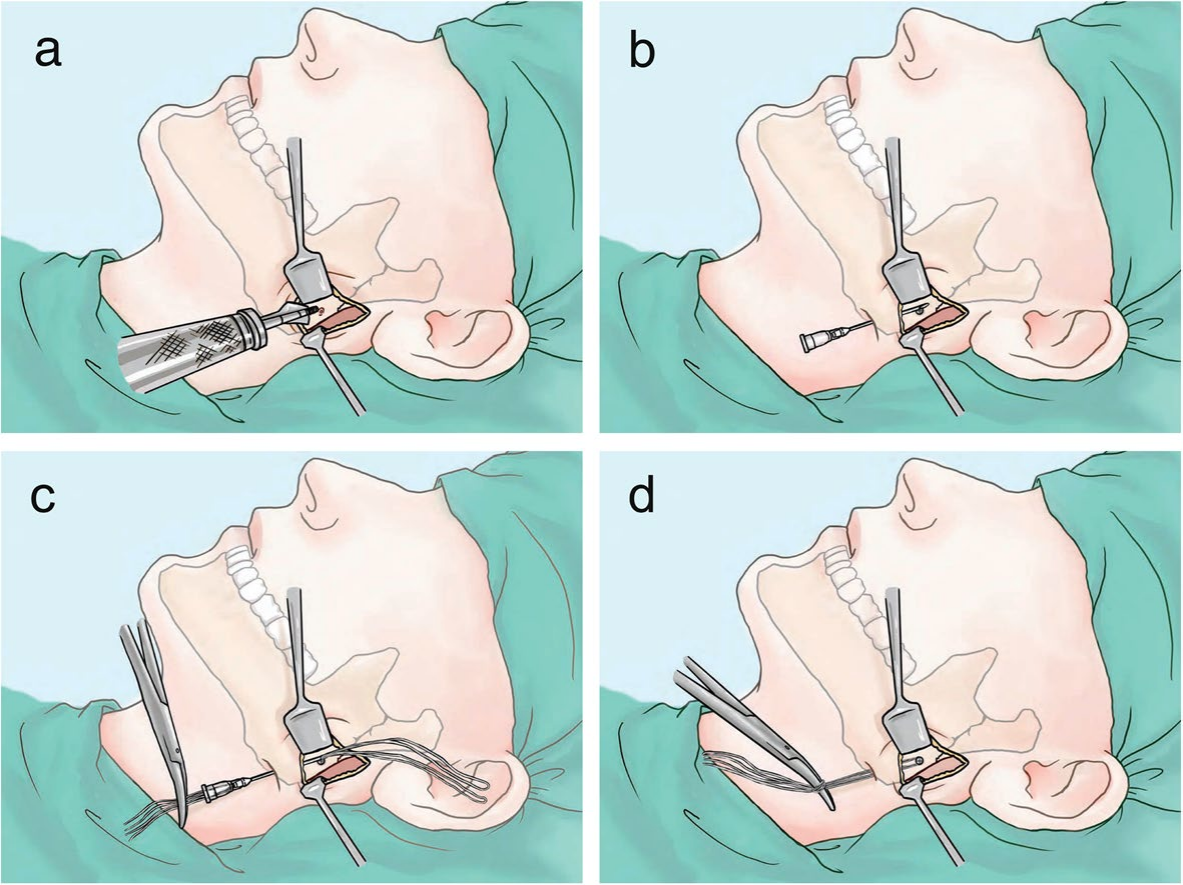
Screw-and-wire technique for condylar fracture reduction. **a** Screw placement on lateral mandibular ramus. **b** Syringe needle insertion upward from mandibular angle. **c** Twisted steel wires threaded through syringe needle. **d** Wire secured to screw with traction via curved clamp
- Anatomical Models: High-fidelity craniofacial models replicated relevant anatomical landmarks such as the facial nerve, lateral pterygoid muscle, and zygomatic arch. The facial nerve was simulated with differently thickened wires wrapped in yellow sheaths, and the lateral pterygoid muscle attachment was modeled using springs, aiding visualization and confidence in practicing surgical techniques.
- Hands-On Workshops: Conducted in the surgical skills laboratory, workshops allowed small groups to practice techniques under faculty guidance, reinforcing theoretical knowledge and building practical skills. Real-time feedback ensured skill refinement and confidence building.

To enhance learning, diverse materials were employed:

- Visual Aids: Instructional videos, diagrams, and anatomical charts supplemented demonstrations and practice sessions. Surgical images and diagrams were redrawn when necessary to better illustrate scenarios and details. Figure 2 shows an example schematic diagram of screw and twisted wire traction, highlighting Category II techniques.

### Schedule

**Fig. 3:**
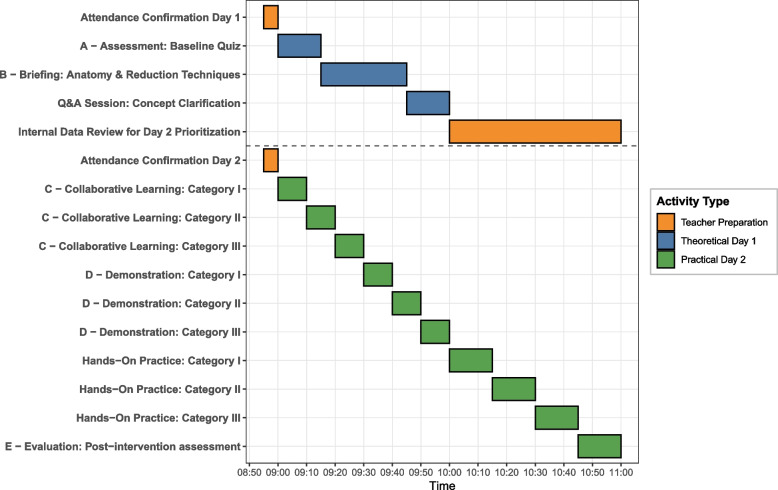
Gantt flowchart of ABCDE framework implementation across Day 1 and Day 2

The educational intervention was structured as a two-day teaching plan, combining theoretical and practical training to comprehensively cover the management of condylar fractures (Fig. 3). Attendance was confirmed through roll calls at the beginning of each session.

- Day 1: Theoretical Foundation
  - Assessment (15 min): Baseline knowledge was evaluated via a validated quiz before any instructional content was delivered. This assessment informed both immediate clarification during the Question & Answer (Q&A) and targeted reinforcement in the practical applications on Day 2.
  - Briefing (30 min): An experienced instructor delivered a foundational lecture supported by schematic diagrams on mandibular anatomy and reduction techniques.
  - Q&A Session (15 min): Interactive Q&A to clarify concepts and enhance understanding.
- Day 2: Practical Application
  - Collaborative Learning (10 min per group): Residents were divided into three groups, each researching and presenting a specific reduction category to their peers.
  - Demonstration (10 min per technique): Faculty demonstrated reduction techniques using anatomical models, emphasizing key steps and potential pitfalls.
  - Hands-on Practice (15 min per group rotation): Residents practiced techniques under supervision, applying collaborative research findings while receiving feedback from instructors. Each group rotated to ensure exposure to all three categories, totaling 105 minutes for collaborative learning and demonstrations.
  - Evaluation (15 min): Post-intervention assessment to evaluate residents’ grasp of theoretical concepts.

### Outcomes

Residents completed a written test (10 multiple-choice questions, 5 points each, total 50 points) before and after the intervention to assess theoretical knowledge. The pre-intervention test corresponded to the Assessment stage and the post-intervention test to the Evaluation stage of the ABCDE framework. Pre-intervention questions focused on basic knowledge, classification, and surgical approaches. Post-intervention questions were embedded in clinical scenarios to assess decision-making and application of knowledge. Scores were not tied to academic credit or rewards.

Prior to the intervention, participants were asked an open-ended survey regarding their initial impressions of the ABCDE framework during the roll call and pre-intervention assessment test on Day 1 (Table 2, Part 1). No additional time was allocated for this item.

**Table 2:** Survey content

Questions	Measures
**Part 1**	
What is your initial reaction to the ABCDE educational framework for teaching condylar fracture reduction?	Open-ended question
**Part 2**	
1. How would you rate your overall satisfaction?	5-point Likert-type scale
2. The ABCDE educational framework has enhanced my learning	5-point Likert-type scale
3. Please describe how the ABCDE framework did or did not enhance your learning. (e.g., learning enthusiasm, learning burden, practical skills, communication, content practicality, or any other aspects.)	Open-ended question
4. I would recommend this educational framework to others	Net promoter score (NPS)
5. Please share your thoughts on how the ABCDE framework in particular made you more or less inclined to recommend it to others. (e.g., practicality in surgical application, clarity of knowledge structure, engagement in teaching, memorability of content, or other aspects related to ease of use.)	Open-ended question

Immediately after the completion of the post-intervention test, residents were administered a five-item post-intervention survey (Table 2, Part 2), which was to be completed within 30 min. The survey included:

- A 5-point semantic differential scale measuring overall satisfaction (“Strongly satisfied” to “Strongly dissatisfied”),
- A 5-point Likert-type scale assessing agreement with the effectiveness of the framework (“strongly agree” to “strongly disagree”),

One NPS item asking participants how likely they would be to recommend the framework to others.

Additionally, two open-ended questions explored learners’ in-depth experiences, particularly focusing on *perceived usefulness* and *perceived ease of use*. These constructs reflect the central ideas of the TAM, where *perceived usefulness* refers to the extent to which learners believe the framework enhances their learning effectiveness, and *perceived ease of use* describes how easily they can engage with and apply the framework in practice. Examples of these questions included:

“Please describe how the ABCDE educational framework did or did not enhance your learning.” (assessing perceived usefulness).

“Please share your thoughts on how the ABCDE framework in particular made you more or less inclined to recommend it to others.” (exploring overall perception and NPS rationale)

These open responses were analyzed qualitatively to complement the quantitative findings and provide richer insights into learners’ acceptance and engagement with the instructional design. Specifically, we examined whether residents perceived the ABCDE framework as enhancing their ability to effectively manage condylar fractures (*perceived usefulness*) and whether they found the framework easy to understand and apply (*perceived ease of use*).

### Statistical analysis

All statistical analyses were conducted using RStudio (Version 2024.12.1 Build 563) with R (version 4.4.3 (2025–02-28 ucrt)). A significance level of p < 0.05 was used for all tests. The data analysis followed a mixed-methods approach. For quantitative assessment, Wilcoxon signed-rank test was applied to compare pre- and post-intervention scores, while Generalized Linear Mixed Models (GLMM) with gamma distribution were used to evaluate the interaction effects of variables. Qualitative data were analyzed through a combination of text mining and manual thematic coding to identify patterns in participant feedback. Here is a breakdown of the detailed procedures and methods employed to achieve the above analyses.

Prior to parametric testing, the normality of continuous variables was assessed using the Shapiro–Wilk test (*stats* package), and homogeneity of variances was evaluated using Levene’s test (*car* package). To assess reliability and internal consistency of Likert-scale items and NPS, Cronbach’s α and exploratory factor analysis were conducted using the *psych* package. For categorical data, correspondence analysis (*FactoMineR* package) was performed to explore associations between variables, with visualizations generated using *factoextra*. Paired comparisons between pre- and post-intervention scores were analyzed using paired t-tests for normally distributed data or Wilcoxon signed-rank tests for non-parametric data (*stats* package). A GLMM was fitted using the *lme4* package to evaluate the impact of training status on outcomes while accounting for potential confounding effects, such as grade level and surgical experience.

Qualitative data from open-ended responses were analyzed through a thematic coding approach that integrates both manual coding and text mining techniques to enhance the efficiency and robustness of the analysis. The process began with collaborative coding sessions involving all investigators to ensure a shared understanding of the detailed themes within residents’ pre- and post-intervention evaluations. The *tidytext* package facilitated thorough text preprocessing by performing tokenization, removal of stop words, and conducting sentiment analysis, before organizing text data into a tidy format. This facilitated downstream visualizations such as Sankey diagrams and heatmaps by using the broader *tidyverse* ecosystem (e.g., *dplyr*, *ggplot2*). Pathways between themes were visualized using Sankey diagrams (*networkD3* package), with flow percentages indicating dominant thematic transitions. To integrate qualitative findings with quantitative outcomes, coded themes were converted into binary matrices via one-hot encoding (*caret* package). Pearson correlation coefficients were calculated, followed by Holm-Bonferroni correction for multiple comparisons (*Hmisc* package), and results were visualized as heatmaps using *ggplot2*.

## Results

### Quantitative analysis

Thirty-nine dental residents participated in the ABCDE educational framework for mandibular condylar fracture reduction training. Of these, 36 residents completed both the pre- and post-intervention tests and the questionnaire, while three attended the program but did not participate in assessments. Post-hoc survey data revealed that 13 residents (34.11%) had prior condylar fracture surgery experience, while 23 (63.89%) had no direct surgical exposure. The basic information of the residents is shown in Table 3.

**Table 3:** Basic information and prior surgical experience in condylar fractures among standardized dental residents (*N* = 36, post-hoc survey data)

	Grade 1	Grade 2	Grade 3
Surgery	2 (5.56%)	6 (16.67%)	5 (13.89%)
Non—Surgery	12 (33.33%)	6 (16.67%)	5 (13.89%)

Of the 39 dental residents enrolled in the study, three residents (*N* = 3) did not complete the survey

### Questionnaire quality and factorial validity

The results show that the questionnaire has good internal consistency and the data is suitable for factor analysis, with a single dominant factor explaining most of the variance, indicating the ABCDE framework’s effectiveness is best represented by a unidimensional construct. Subsequent paragraphs detail the statistical analyses and evidence.

The reliability of the questionnaire was confirmed using Cronbach’s α coefficient (α = 0.84), indicating robust internal consistency. The Kaiser–Meyer–Olkin (KMO) measure (KMO = 0.71) and Bartlett’s test of sphericity (*p* < 0.001) further validated the suitability of the data for factor analysis. Parallel analysis revealed that the first eigenvalue (2.17 for original factors and 2.43 for principal components) exceeded those of subsequent factors, suggesting a single dominant factor explaining 40% of the variance. Subsequent eigenvalues were statistically negligible, implying that additional factors lacked meaningful explanatory power.

To refine the factor structure, exploratory factor analysis (EFA) was performed using the minimum residual (minres) method with varimax rotation, initially specifying three factors. The results demonstrated that the first factor (MR1) accounted for 40% of variance, while the second (MR2, 32%) and third (MR3, negligible) factors were secondary or noise-driven. The model exhibited an excellent fit (Tucker-Lewis Index, TLI = 1.06; root mean square residual, RMSR = 0.00).

All variables (satisfaction, enhancement, and NPS) strongly correlated with MR1, aligning with its interpretation as a unidimensional construct reflecting the ABCDE framework’s core effectiveness. The secondary factors (MR2 and MR3) did not contribute meaningfully to the model, suggesting that the framework’s impact is best captured by a single latent dimension encompassing teaching quality, learner satisfaction, and advocacy.

### Correspondence analysis

The analysis revealed consistent associations: high satisfaction predicted advocacy (NPS), and perceived learning enhancement strongly correlated with both satisfaction and recommendation behavior. These findings support the TAM model, suggesting that *perceived usefulness* (learning enhancement) and *perceived ease of use* (satisfaction) are interrelated and jointly influence learners’ willingness to recommend the framework. Subsequent paragraphs detail the statistical analyses and evidence.

**Fig. 4:**
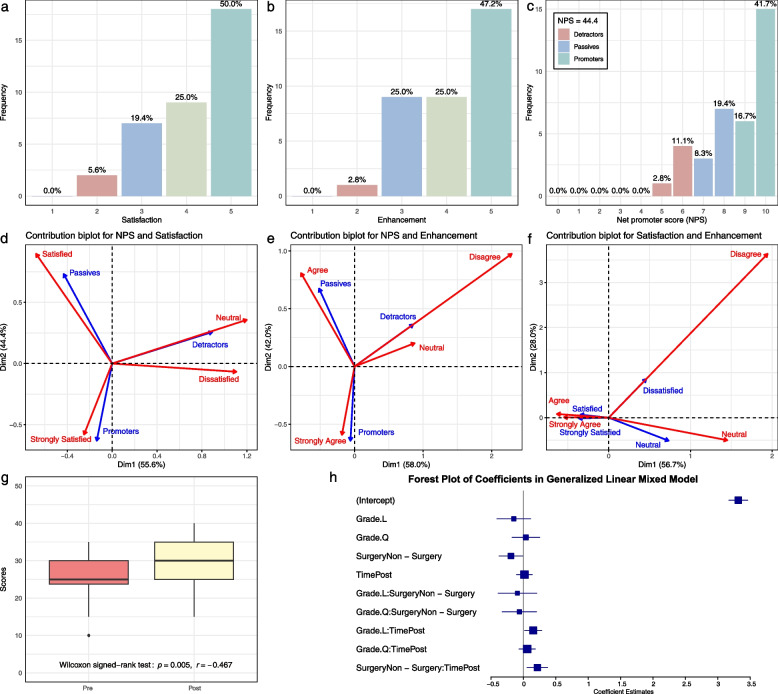
Quantitative outcomes of educational intervention. **a–c** Resident-reported ratings for (**a**) satisfaction, (**b**) perceived learning enhancement, and (**c**) Net Promoter Score (NPS). **d–f** Contribution biplot from correspondence analysis showing relationships among NPS categories, satisfaction levels, and perceived learning enhancement. Point proximity indicates association strength, while distance from the origin reflects contribution to principal dimensions. **g** Median performance scores pre- and post-intervention with 95% CI [pre: 25.00 (23.75, 30.00); post: 30.00 (25.00, 35.00)]. Wilcoxon signed-rank test indicated significant improvement (r = −0.47, *p* < 0.01). **h** Forest plot from a generalized linear mixed model (GLMM) showing predictors of score improvement. Fixed effects include time (pre/post), grade level, and surgical experience. Interactions between time and grade, as well as time and surgical experience, were statistically significant (*p* < 0.05)

Figure 4a and b show that over 70% of residents reported positive ratings for satisfaction and learning enhancement, respectively. Figure 4c presents a NPS of 44.44, indicating moderate-to-high advocacy. Fisher’s exact test confirmed significant associations (*p* < 0.001) among NPS, satisfaction, and learning enhancement. Correspondence analysis further clarified these relationships:

Satisfaction and NPS (Fig. 4d): Promoters and “Strongly Satisfied” residents clustered closely along the first dimension, confirming that high satisfaction aligns with advocacy. Neutral/Satisfied residents were more dispersed, contributing less to NPS. Detractors/Dissatisfied residents formed a distinct group.

Learning Enhancement and NPS (Fig. 4e): Promoters and residents who “Strongly Agreed” that the framework improved their skills clustered tightly, indicating a strong link between perceived learning gains and advocacy. Neutral/Agree responses formed a secondary cluster, while Detractors/Disagree responses remained separate.

Satisfaction and Learning Enhancement (Fig. 4f): A strong interdependence was observed between “Strongly Satisfied” and “Strongly Agree” residents, who clustered closely. Satisfied/Agree responses formed a weaker cluster, while Neutral/Dissatisfied/Disagree responses were more dispersed.

### Educational framework effects and associated factors

The results supported the effectiveness of the ABCDE framework in improving knowledge application among medical students, with greater benefits seen in higher-grade students and those without prior surgical experience. Subsequent paragraphs detail the statistical analyses and evidence.

We used a combination of nonparametric and mixed-effects modeling approaches to assess the impact of the ABCDE educational framework on student performance. Given the non-normal distribution of pre- to post-intervention score differences, we conducted a Wilcoxon signed-rank test. Results showed a statistically significant improvement in median test scores, from 25.00 [95% confidence interval (CI): 23.75, 30.00] before the intervention to 30.00 [95% CI: 25.00, 35.00] after the intervention, with a large effect size (r = −0.47, p < 0.01), indicating that the framework had a meaningful positive effect on learning outcomes (Fig. 4g).

To further examine how individual factors such as grade level and prior surgical experience influenced this effect, we fitted a GLMM with Gamma distribution. The model was estimated using maximum likelihood with Laplace approximation, yielding an Akaike Information Criterion (AIC) of 459.84 and a log-likelihood of −217.92, suggesting good overall fit.

Fixed effects analysis (Fig. 4h) revealed:

- A significant intercept (*p* < 0.05), indicating a stable baseline prediction;
- Students without surgical experience scored higher at baseline than those with surgical experience “SurgeryNon − Surgery” (*p* = 0.04);
- A significant interaction between linear grade trends and time “Grade.L:TimePost” (p = 0.02) suggested that students at higher grade levels experienced greater improvement after the intervention;
- A significant interaction between surgical experience and time “SurgeryNon − Surgery:TimePost” (*p* < 0.01) indicated that students without surgical background demonstrated more pronounced score gains following the intervention;
- Other interactions, including quadratic grade trends and main time effects, were not statistically significant (*p* > 0.05).

Random effects analysis showed:

- A residual mean was close to zero (0.0171), suggesting that the model’s predictions aligned well with observed data.
- A relatively high residual standard deviation (0.889) indicates considerable variation in individual learning outcomes. These findings underscore the need for future research to investigate how factors such as motivation, study habits, and prior knowledge may influence learners’ responses to the educational intervention.

### Qualitative analysis

#### Thematic coding and text mining integration

Qualitative data from open-ended responses were analyzed through a thematic coding approach that integrates both manual coding and text mining techniques to enhance the efficiency and robustness of the analysis. This combined approach is justified by the suitability of open-ended survey responses for text mining [33], as well as the systematic nature of thematic analysis, following Braun and Clarke’s framework [34]. The manual coding approach was inspired by the collaborative coding methodology described by previous study [23], which emphasized investigator consensus and thematic fidelity.

To ensure methodological transparency and reproducibility, we followed a structured process beginning with text preprocessing. Text preprocessing, including tokenization, removal of stop words, and conducting sentiment analysis, was performed using the *tidytext* package in R. This step ensured that the textual data was clean, consistent, and ready for further qualitative and quantitative analyses. The resulting tidy format also allowed seamless integration with the broader *tidyverse* ecosystem (e.g., *dplyr*, *ggplot2*) for visualization and statistical exploration.

The insights derived from text mining, such as word frequency distributions and sentiment trends, informed the initial coding framework and helped identify potential thematic patterns. This data-driven foundation enhanced the rigor and objectivity of the subsequent manual coding process.

Investigators then engaged in iterative discussions to identify salient themes and inflection points tied to the ABCDE educational framework. For each resident’s response, codes were selected with precision to capture thematic content without overinterpretation. Existing codes were applied when responses aligned with predefined themes, while new codes were created ad hoc for emergent themes when no existing code was applicable. Ambiguous phrasing was addressed through strict textual fidelity, avoiding speculative interpretations unless consensus was reached among all investigators.

Throughout the coding process, we maintained alignment with the theoretical constructs of the ABCDE educational model. This ensured that the identified themes were not only descriptive but also contextually meaningful within the framework of our intervention. By grounding the thematic development in this pedagogical model, we enhanced the interpretability and relevance of the findings in relation to the intended educational outcomes.

#### Visualizing thematic pathways with Sankey diagrams

The main finding is that most residents who started with *Active Exploration* and *Interest Driven* learning eventually found the program highly practical, leading them to become promoters.

Sankey diagrams were created using the *networkD3* package in R, which visually represents the flow between different categories of resident evaluations. Each node represents a category, and the width of the links corresponds to the frequency of transitions between categories. The diagram visualizes sequential transitions among thematic categories derived from the manual coding of open-ended responses. Each node represents a distinct thematic category (e.g., “*Active Exploration*”, “*Interest Driven*”, “*High Practicality*”, “*Promoter*”), while the links between nodes represent the flow of responses across these categories.

By mapping qualitative themes onto measurable flows, the Sankey diagram provides a holistic view of dominant and emergent patterns in residents’ feedback, making it particularly useful for identifying key drivers of satisfaction (e.g., *perceived usefulness*) as well as anomalous or unexpected evaluation trajectories.

**Fig. 5:**
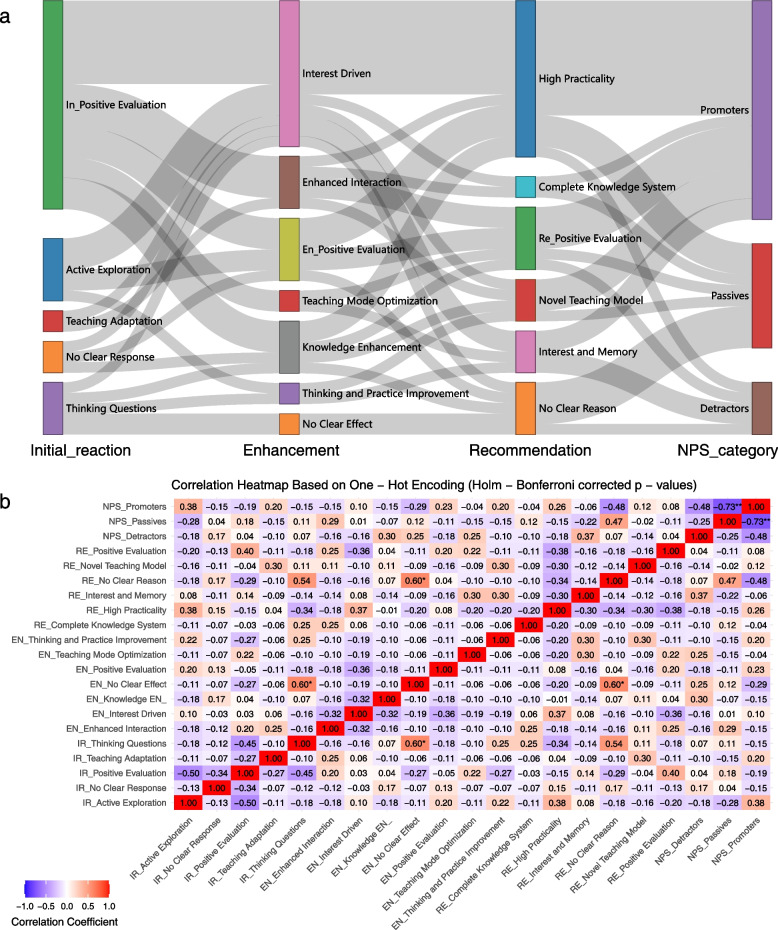
Qualitative feedback analysis. **a** Sankey diagram depicting thematic flow and connections in residents’ open-ended feedback. Width of bands corresponds to frequency of thematic linkage. Themes were derived through inductive coding of qualitative responses. **b** Heatmap displaying pairwise correlations between key themes identified in qualitative feedback. Darker colors indicate stronger positive or negative associations

The Sankey diagram visualizing the flow across stages (Initial Reaction → Enhancement → Recommendation → NPS Category) revealed no obvious anomalous paths in the data. All observed pathways exhibited logical consistency with the expected process flow, and no unexpected or contradictory transitions were detected between stages. This consistency strengthens the reliability of the coding framework and the validity of insights derived from the qualitative data. Sankey diagrams (Fig. 5a, Supplementary Material 1) visualized the core transformation pathways in resident evaluations:

Primary Pathway: *Active Exploration → Interest Driven → High Practicality → Promoters*. This pathway accounted for 8.3% (3/36) of total flow, validating the hypothesis that perceived practicality drives satisfaction (Supplementary Material 2). Key findings included:

Approximately 73.3% (11/15) of *High Practicality* assessments directly translated into Promoters, underscoring the critical role of practicality in boosting the NPS.

*Interest Driven* emerged as the dominant theme (38.9%, (14/36)) in learning outcomes (Enhancement), with 50% (3/6) of *Active Exploration* responses transitioning to this category, highlighting the efficacy of interactive teaching designs in motivating learners.

While a marginal trend was observed between recommendation reasons and NPS categories (p = 0.054), the association did not reach statistical significance, which may be attributable to limited sample size.

#### Quantitative analysis of categorical themes

The results showed that students who raised questions about the teaching framework before training (*IR_Thinking Questions*) were significantly more likely to simply write “no” without further explanation when asked about the reasons for enhancement learning after training (*EN_No Clear Effect*) and the reasons for recommendation (*RE_No Clear Reason*). Heatmap analysis and correlation coefficient calculations both confirmed this significant positive association, indicating a strong link between initial doubts and subsequent lack of detailed explanations. Subsequent paragraphs detail the statistical analyses and evidence.

Categorical variables were converted to numerical format using one-hot encoding, a method that transforms qualitative categories into a binary matrix format suitable for statistical analysis. Each category is represented as a separate binary variable (dummy variable), where a value of 1 indicates the presence of that category and 0 indicates its absence. This approach avoids imposing any artificial ordinal relationship between categories while enabling their use in correlation analyses.

For example, thematic codes such as “*EN_No Clear Effect*”, “*IR_Thinking Questions*”, and “*RE_No Clear Reason*” were originally derived from open-ended responses and existed as non-numeric categories. One-hot encoding transformed these into binary indicators, allowing us to quantify their co-occurrence across responses and analyze them using standard statistical techniques.

Following this transformation, Pearson correlation coefficients were calculated to assess the strength and direction of linear relationships between different encoded thematic categories. To account for multiple comparisons and reduce the risk of Type I errors, p-values were adjusted using the Holm-Bonferroni method.

Heatmap analysis (Fig. 5b) revealed significant positive correlations between *EN_No Clear Effect* and *IR_Thinking Questions* (r = 0.60, p < 0.05) and *RE_No Clear Reason* (r = 0.60, p < 0.05), suggesting that ambiguous learning outcomes may be linked to reflective questions or unclear recommendation reasons.

To assess monotonic associations with the ordered NPS categories (Detractors < Passives < Promoters), NPS was converted to numerical ranks (NPS_rank: 1–3), while other variables remained one-hot encoded. Spearman’s correlation coefficients for *EN_No Clear Effect* with *IR_Thinking Questions* (ρ = 0.60, p < 0.05) and *RE_No Clear Reason* (ρ = 0.60, p < 0.05) matched the Pearson results exactly (Supplementary Material 3). This analysis reinforces the consistency of findings across correlation methods (Pearson for linear co-occurrence vs. Spearman for monotonic trends), with identical effect sizes reflecting the binary nature of encoded variables.

## Discussion

The ABCDE framework demonstrates robust efficacy in enhancing dental residents’ knowledge acquisition, skill mastery, and clinical confidence in managing mandibular condylar fractures. Quantitative results, including a statistically significant improvement in post-intervention test scores (median increase from 25.00 to 30.00, p < 0.01) and a NPS of 44.44, validate its practical use and learner acceptance. These findings align with the TAM, where *perceived usefulness* (e.g., *Interest Driven* of case-based surgical atlases) and *perceived ease of use* (e.g., *High Practicality* of hands-on workshop designs) drive engagement and advocacy. The GLMM further revealed that learners without prior surgical experience benefited most from the framework, underscoring its potential to address experiential gaps in novice training through structured, hands-on practice and real-time faculty feedback. Findings from qualitative analysis indicate that the strong correlation between *Interest Driven* engagement and *Active Exploration* (50% conversion) underscores the role of interactive teaching in fostering intrinsic motivation. This resonates with studies demonstrating that curiosity-driven learners benefit disproportionately from hands-on activities and reflective questioning [35].

The ABCDE framework’s modular, learner-centered design complements CBL by balancing structured guidance with active problem-solving. While CBL encourages active engagement through open-ended clinical scenarios [25], the ABCDE framework provides a scaffolded sequence of phases—Assessment, Briefing, Collaborative Learning, Demonstration, and Evaluation—that ensures progressive skill development. For example, the Briefing phase incorporated case-based surgical atlases to illustrate mandibular anatomy and reduction techniques, functioning as guided case-embedded didactics rather than pure CBL. These visual aids contextualized theoretical concepts within real-world surgical scenarios, enhancing clinical relevance and procedural understanding without requiring learners to independently formulate diagnoses or treatment plans. This approach aligns with Gagné’s nine events of instruction [36], particularly the phases of gain attention and presenting content, where visual and contextual examples anchor abstract concepts.

The ABCDE framework aligns with well-established pedagogical models such as the flipped classroom and problem-based learning (PBL), both of which emphasize critical thinking and skill application over passive knowledge acquisition [37]. Like the flipped classroom, ABCDE separates knowledge acquisition from skill practice; however, it differs in implementation. While the flipped classroom typically shifts content delivery to pre-class activities such as video lectures, readings, or online modules [38], ABCDE integrates case-based surgical atlases and schematic diagrams directly into the Briefing phase. This ensures that theoretical concepts are introduced within authentic clinical contexts, maintaining the benefits of contextual learning while minimizing reliance on digital tools, making the framework especially suitable for resource-limited settings.

In contrast to the flipped classroom’s emphasis on learner autonomy, ABCDE provides scaffolded guidance through expert demonstrations and real-time feedback, ensuring procedural accuracy in high-stakes surgical training. This “from guided practice to independent mastery” structured progression aligns with both Vygotsky’s zone of proximal development [39] and Kolb’s experiential learning theory [40], which emphasize the importance of concrete experiences and active experimentation in skill retention. For example, the Collaborative Learning and Demonstration phase promotes peer-led case discussions akin to those in a flipped classroom, but with faculty oversight to ensure clinical safety and instructional coherence.

Unlike PBL, which prioritizes self-directed inquiry [41], ABCDE offers clear procedural benchmarks, making it more accessible to junior residents who require structured guidance. This contrast reflects the framework’s alignment with Miller’s Pyramid of Clinical Competence [42, 43], addressing all levels from theoretical knowledge (“Knows”) to autonomous application (“Does”).

Throughout this process, faculty teachers played a crucial role by facilitating discussions, providing real-time feedback, and addressing difficulties, highlighting the importance of educators in creating an environment where students feel confident to engage in active learning techniques [44]. A critical strength of the ABCDE framework lies in its integration of formative assessment to guide instructional adaptation, which enhances its responsiveness to learner needs while maintaining structured progression. For example, the baseline assessment administered prior to the Day 1 lecture served not merely as a diagnostic tool but as a pedagogical lever to inform real-time and subsequent instructional adjustments:

During the Q&A session following the lecture, instructors systematically reviewed commonly missed items to address persistent misconceptions and align foundational knowledge before advancing to practical training. This immediate feedback loop ensured that learners progressed to skill application with a shared conceptual baseline.

Post-Day 1 analysis of quiz results identified recurring knowledge gaps (e.g., challenges in anatomical landmark identification or screw placement techniques). These insights were then operationalized to tailor Day 2 sessions, where high-fidelity models, visual aids, and step-by-step procedural breakdowns were strategically deployed to reinforce areas of difficulty.

Meanwhile, the instructor feedback and clear connections to summative assessment could significantly increase student engagement and learning [45]. This iterative use of assessment aligns with formative assessment principles, where data-driven adjustments optimize learning outcomes without disrupting instructional pacing [46].

The effectiveness of the ABCDE framework can be understood within the context of broader educational theories. In contrast to the ADDIE (Analyze, Design, Develop, Implement, Evaluate) model [47], which is a macro-level curriculum design approach structured around sequential phases, the ABCDE framework functions as a micro-level, skill-specific intervention that is particularly well suited for short-term training workshops. ADDIE’s upfront needs analysis is complemented by ABCDE’s iterative refinement during demonstration and evaluation phases, making it particularly suitable for niche techniques like condylar fracture reduction.

The ABCDE framework demonstrates broad adaptability across surgical disciplines and educational contexts. It employs low-fidelity simulation tools like anatomical models and case scenarios, making it suitable for resource-limited settings, while its modular design allows integration with advanced technologies like VR in well-equipped institutions. For example, the Demonstration phase can be enhanced with VR-guided tutorials, and Collaborative Learning can facilitate interprofessional discussions across surgical specialties. This flexibility aligns with the TAM, where perceived usefulness and ease of use are key drivers of learner engagement and adoption.

Rooted in both TAM principles and pedagogical pragmatism, the framework emphasizes clinical relevance (e.g., through case-based post-tests) and intuitive design (e.g., structured workshop rotations), fostering intrinsic motivation and skill retention, and it highlights its ability to “bridge book knowledge and surgery room reality” and “build confidence through repeated, guided practice”, directly addressing the persistent theory–practice gap in surgical education.

Mnemonics are well-established educational strategies that have proven useful in encoding, retention and retrieval [48]. And acronym mnemonics are beneficial, particularly for the learning of a sequential procedural task [49]. While the primary focus of this study is the ABCDE framework as an instructional strategy rather than a mnemonic device, it is important to acknowledge its conceptual roots in the broader use of ABCDE acronyms in medicine. In clinical domains, ABCDE serves as a cognitive scaffold for rapid decision-making. In contrast, the ABCDE framework operationalized in this study serves not merely as a memory aid, but more importantly as a pedagogical tool designed to organize instructional phases and promote skill mastery.

To clarify this distinction and avoid confusion with clinical mnemonics, we summarize the various applications of the ABCDE framework in medical education and practice in Table 4, based on studies reported in references [50–65]. This comparison highlights how the framework’s educational purpose diverges from its clinical counterparts while retaining the ABCDE acronym’s intuitive structure for learner accessibility.

**Table 4:** Some ABCDE mnemonics in medicine

No	ABCDE	Application	References
1	**A**ssessment, **B**riefing, **C**ollaborative learning, **D**emonstration, **E**valuation	Educational framework designed to integrate theoretical knowledge with practical skills	This study
2	plan **A**head using implementation science, **B**e clear and thoroughly describe the intervention by using the TidIER checklist, use a **C**hecklist to comprehensively report study components, select a study **D**esign carefully, assess **E**ffectiveness and implementation by selecting meaningful outcomes	Five steps to high quality antimicrobial stewardship research	[50]
3	**A**symmetry, **B**order, **C**olor, **D**iameter, **E**volving	Melanoma: Diagnosis and Treatment	[51]
4	**A**dvocacy, **B**randing, **C**ommunication, **D**irect networking, **E**ducation	An approach for physicians to use social media for professional purposes	[52]
5	**A**dvanced cancer; potential **B**enefits; **C**linical conditions and risks; **D**esire, values, preferences and beliefs; prognostic **E**stimation	Suggested therapeutic decision-making process in patients with far advanced cancers	[53]
6	**A**void shaming/personal opinions, **B**uild a rapport, **C**hoose a communication approach, **D**evelop a debriefing content, **E**nsure the ergonomics of debriefing	A simplified model for debriefing during simulation in emergency medicine	[54]
7	**A**cknowledge; **B**ear witness; offer **C**oping support; **D**ebrief; **E**nlighten, Engage, and Educate	An approach to thwart early triggers of symptoms on the burnout continuum for trauma surgeons	[55]
8	**A**nthropometry, **B**iochemistry, **C**linical, **D**ietary intakes, **E**nvironment and evaluation	A brief approach to nutritional assessment in preterm infants	[56]
9	**A**ssess, **B**alance, **C**omplications, **D**MDs (Diabetes-/disease-modifying drugs), **E**xposure	Systematic evaluation of patient’s diabetes status during the peri-discharge period to prevent subsequent hospitalization	[57]
10	**A**ge, **B**ody weight, **C**omplications and Comorbidities, **D**uration of diabetes, life **E**xpectancy & Etiology	Five different clinical factors considered before personalized approach for type 2 diabetes pharmacotherapy	[58]
11	**A**nkle-brachial index, **B**-lines, **C**arotid intima media thickness, **D**iameter of the abdominal aorta and of the inferior cave vein, **E**chocardiographic assessment of the ejection fraction	Five-step vascular ultrasound examination in heart failure	[59]
12	**A**ge, **B**ilirubin, **C**ommon bile **d**uct diameter, **E**ndoscopic retrograde cholangiopancreatography	The ABCdE score for predicting lithotripsy assistance during transcystic bile duct exploration by laparoendoscopy	[60]
13	**A**synergy, **B**-lines, **C**ontractile reserve based on force, **D**oppler-based coronary flow velocity reserve in left anterior descending coronary artery, **E**KG-based heart rate reserve	Stress echocardiography with ABCDE protocol	[61]
14	**A**ssess, prevent, and manage pain; **B**oth spontaneous awakening trials and spontaneous breathing trials; **C**hoice of analgesia and sedation; **D**elirium: assess, prevent, and manage; **E**arly mobility and exercise; **F**amily engagement and empowerment	The ABCDEF bundle in critical care	[62]
15	I **A**m, **B**elong, **C**an, **D**read, **E**xist	Presentations of self in clinical encounters	[63]
16	**A**irway, **B**reathing, **C**irculation, **D**isability, **E**xposure & Environment	Initial assessment and treatment in all clinical emergencies	[64]
17	**A**dvance preparation, **B**uilding a therapeutic relationship, **C**ommunicating well, **D**ealing with patient and family reactions, **E**ncouraging/validating emotions	How physicians break bad news to patients	[65]

### Limitations

Our study has three main limitations: 1) The near-significant association between recommendation reasons and NPS categories (p = 0.054) necessitates larger-scale validation; 2) While the GLMM controlled for individual differences, unexamined variables like learning preferences motivation require exploration to optimize framework adaptability; 3) Qualitative findings were limited to self-reported responses potentially influenced by social desirability effects. Three critical priorities for further investigation include assessing the framework’s scalability in diverse clinical/resource contexts, evaluating sustained competency development in novice learners, and conducting comparative effectiveness trials against conventional training paradigms.

## Conclusions

The ABCDE framework presents a structured, theory-driven educational model that effectively bridges theoretical knowledge and practical skill in condylar fracture reduction education, with median test scores improving from 25.00 to 30.00 (*p* < 0.01), a NPS of 44.44, and over 70% resident satisfaction. Rooted in TAM, its modular design (Assessment-Briefing-Collaborative Learning-Demonstration-Evaluation) prioritizes perceived usefulness and ease of use, addressing conventional teaching limitations. Compatible with VR and scalable to other surgical fields, the framework warrants future study on long-term retention and cross-disciplinary efficacy.

## Supplementary Information

Supplementary Material 1.

Supplementary Material 2.

Supplementary Material 3.

## Data Availability

The datasets used and/or analysed during the current study are available from the corresponding author on reasonable request.

## Abbreviations

CA: Correspondence analysis
CBL: Case-Based Learning
CI: Confidence interval
GLMM: Generalized Linear Mixed Model
KMO: Kaiser–Meyer–Olkin
NPS: Net Promoter Score
OMFS: Oral and maxillofacial surgery
PBL: Problem-based learning
TAM: Technology Acceptance Model
TMJ: Temporomandibular joint
VR: Virtual reality
