# Supplementary figures and images for: Assessing effectiveness of ABCDE Framework for teaching condylar fracture reduction in dental education: a mixed methods study
Source: BMC Med Educ. 2025 Jul 31;25:1129. doi: 10.1186/s12909-025-07705-7 (PMC12312604; doi:10.1186/s12909-025-07705-7)

Correlation Heatmap with Ordered NPS (Holm–Bonferroni Corrected)

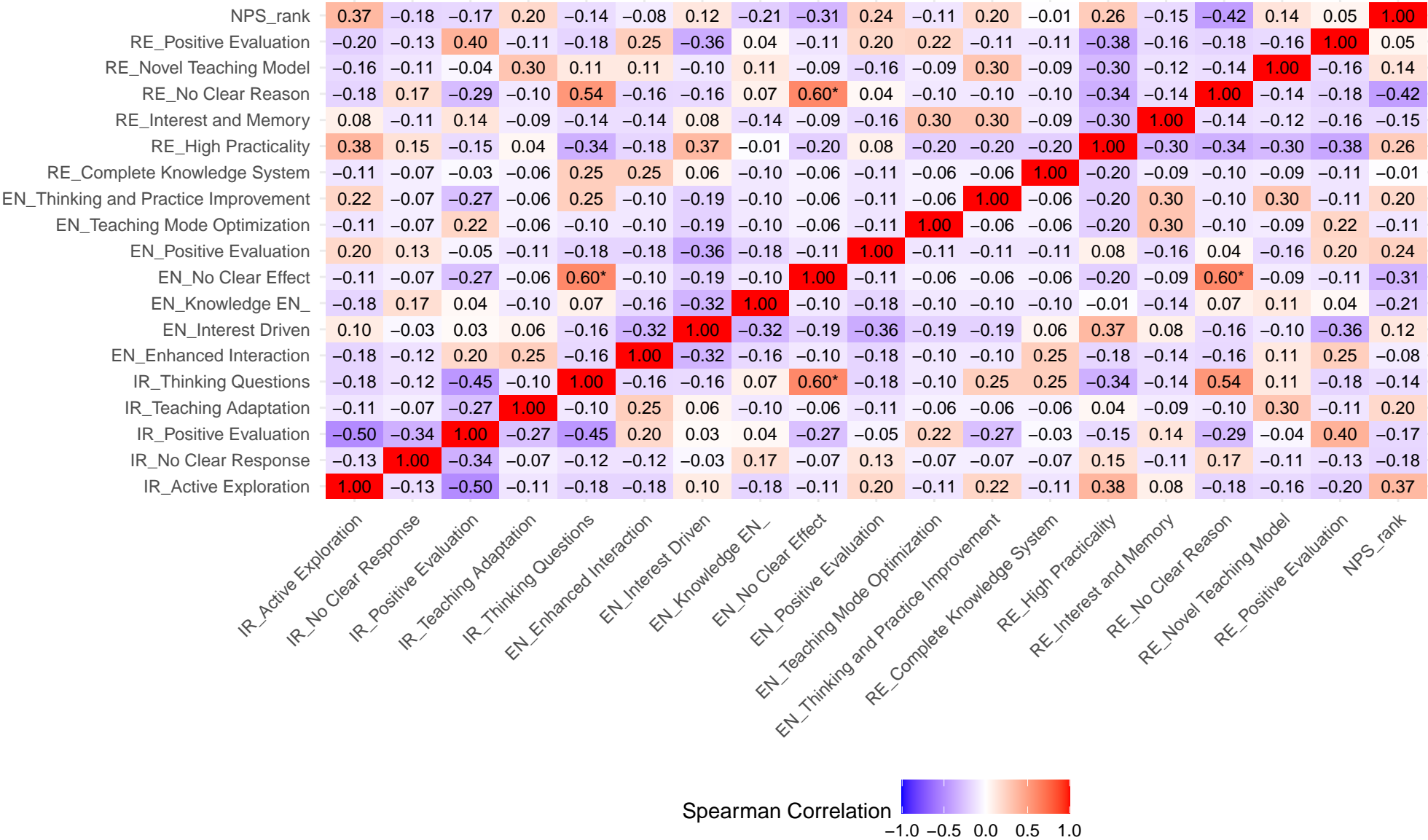

Supplement: Supplementary file 3 — Supplementary Material 3. [file 12909_2025_7705_MOESM3_ESM.pdf]
